# A Singular Extenuation in the Leg, with the Method of Cure

**Published:** 1803-03-01

**Authors:** James Lucas

**Affiliations:** Conisbrough, Member of the College of Surgeons in London


### *A singular* Extenuation *in the* Leg, *with the Method of Cure; by* Mr. James Lucas, *of Conisbrough, Member of the College of Surgeons in London.*

A Young gentleman, free from any other malady, when on business in Ireland, that obliged him to stand and walk much every day, found his left leg become gradually weaker than the right, yet without any further pain than such as might be attributed to all the muscles being affected by the uncommon exertion required. He soon became unequal to transact his business, and discovered, by admeasurement, that his leg was pining away, which induced him to go to Dublin, where he remained some weeks under the management of a surgeon of eminence, but without any perceptible relief. He then revisited England, and placed himself under the care of a late hospital surgeon in London, for as long a time, without experiencing any greater benefit. As those whom he had consulted, ascribed the disorder merely to weakness, he was advised to try country air; he accordingly came into Yorkshire, and requested that I would spare no exertion in ascertaining the source of a complaint, which totally disabled him from prosecuting not only important but pressing business.

In fulfilling his anxious wishes, the wasting or atrophy of the affected limb appeared to me not to be universal, but confined to the fore part of the leg, and along the course of the tibialis anticus muscle; I therefore desired that his foot might be denuded, in order that I might trace the line of the muscle throughout. At its insertion into the cuneiform bone there was an evident intumescence and induration, but without tenderness or inflammation. As it had never been painful, the gentleman had never supposed it to be of any consequence, nor had it ever been inquired after, or seen by the other surgeons.

To the best of his recollection there had been some fulness in that part of his foot ever since it had been much bruised by a fall from a horse, but though it had increased, it had done so imperceptibly. Knowing that my patient was an able artist in drawing, I offered to explain my notion of his complaint by a muscular table in Albinus. In this explication I encouraged him to hope, that, by discussing the swelling, the whole emaciated muscle, from admitting customary nutrition, would recover its action, plumpness, and strength.

By covering the affected part with a soap cerate plaster, and every night rubbing in half a drachm of the ung. hydrarg. the tumour disappeared in a few weeks. Clutton's febrifuge spirit was twice a day applied to the wasted part of the limb, which was also rubbed with a flesh-brush, and exercised.

Within two months a cure was completed, nor did the complaint ever return. It may be questioned, whether the extenuation had not increased from the limb having been preserved, both in Dublin and London, chiefly in a state of rest.

When from a dislocation, or any other cause, a limb is weak, inactive, and emaciated, local exercise highly contributes to the cure; and upon such principle, I have recommended bandages with elastic wires, to give motion, as well as support, to the affected parts. (See London Medical Journal, Index, vol. xi.)

The source of diseases may not, when first seen, be as clear as afterwards, which may call for repeated investigation to prevent a latent cause being overlooked. As men of eminence, from their accustomed intuitive skill, sometimes risk hasty and incautious decisions, their failure should rather animate less experienced juniors not to be dismayed, but to exert their utmost abilities. As the linimentum bituminis ammoniatum of the Pharm. Chirurg. will sometimes remove ganglions, I should esteem it a likely remedy for discussing a similar intumescence, should the ung. hydrarg. fail or be deemed ineligible. Cold bathing, electricity, and various embrocations, had been applied to the leg, on the inefficacy of which it appears to be unnecessary to enlarge, as it clearly depended on the seat of the malady not having been ascertained.

*January* 30, 1803.

P. S. The case I communicated to Mr. Pearson, in your 7th vol. p. 49, remained well, and the health was re-established. The young lady lately died of a fever.

